# Effect of Dual Al_2_O_3_ MIS Gate Structure on DC and RF Characteristics of Enhancement-Mode GaN HEMT

**DOI:** 10.3390/mi16060687

**Published:** 2025-06-07

**Authors:** Yuan Li, Yong Huang, Jing Li, Huiqing Sun, Zhiyou Guo

**Affiliations:** 1 Institute of Semiconductor Science and Technology, South China Normal University, 55 Zhongshan Avenue, Tianhe District, Guangzhou 510631, China; liamly@m.scnu.edu.cn (Y.L.); sunhq@scnu.edu.cn (H.S.); 2 Guangdong Industrial Training Center, Guangdong Polytechnic Normal University, Guangzhou 510665, China; hy.scut@163.com; 3 School of Information and Engineering, Nanjing XiaoZhuang University, Nanjing 211171, China; jingl@njxzc.edu.cn

**Keywords:** GaN HEMT, Al_2_O_3_ dielectric, MIS structure, TCAD simulation

## Abstract

A dual Al_2_O_3_ MIS gate structure is proposed to enhance the DC and RF performance of enhancement-mode GaN high-electron mobility transistors (HEMTs). As a result, the proposed MOS-HEMT with a dual recessed MIS gate structure offers 84% improvements in cutoff frequency (f_T_) and 92% improvements in maximum oscillation frequency (f_max_) compared to conventional HEMTs (from 7.1 GHz to 13.1 GHz and 17.5 GHz to 33.6 GHz, respectively). As for direct-current characteristics, a remarkable reduction in off-state gate leakage current and a 26% enhancement in the maximum saturation drain current (from 519 mA·mm^−1^ to 658 A·mm^−1^) are manifested in HEMTs with new structures. The maximum transconductance (g_m_) is also raised from 209 mS·mm^−1^ to 246 mS·mm^−1^. Correspondingly, almost unchanged gate–source capacitance curves and gate–drain capacitance curves are also discussed to explain the electrical characteristic mechanism. These results indicate the superiority of using a dual Al_2_O_3_ MIS gate structure in GaN-based HEMTs to promote the RF and DC performance, providing a reference for further development in a miniwatt antenna amplifier and sub-6G frequencies of operation.

## 1. Introduction

The discovery and research of third-generation semiconductors have brought revolutionary changes to the development of 5G communication technology. With communication frequency bands shifting to higher frequencies, technologies such as full-spectrum access [1], massive MIMO [2], and carrier aggregation [3] place new demands on the performance and design of radio frequency (RF) devices and power devices. The unique property of GaN materials is their wide bandgap [4,5], which allows them to maintain a high breakdown voltage even at maximum operating temperatures [6]. GaN has a larger bandgap, higher electron mobility, and higher electron saturation velocity [7,8]. These material properties have a significant impact on the performance of radio frequency devices, while their radiation resistance and high-temperature tolerance ensure stability during application [9,10]. Therefore, GaN is highly suitable for the high frequency and millimeter-wave region, meeting both performance and compact size requirements [11]. In addition to the inherent advantages of GaN, its growth and fabrication process is relatively straightforward. It can be epitaxially grown on heterogeneous substrates such as Si and SiC using Metal-Organic Chemical Vapor Deposition (MOCVD) [12], enabling large-scale mass production capabilities.

The introduction of a dielectric layer between the Schottky gate and the semiconductor forms an MIS (Metal–Insulator–Semiconductor) structure, which can reduce the gate current [13], increase the breakdown voltage [14], and enable a wide gate bias range [15]. Liu et al. reported a high-performance normally-off Al_2_O_3_/AlN/GaN MOS-HEMT exhibiting a maximum drain current of 660 mA/mm, a field-effect mobility of 165 cm^2^/V·s, and a high on/off drain current ratio of 10^10^ [16]. A gate dielectric with a higher permittivity can increase the gate capacitance, provide better control over the channel charge, and also facilitate the reduction in device size [17]. A commonly used gate dielectric, Al_2_O_3_, with a dielectric constant of nine, can be fabricated by depositing Al on a nitride material surface and re-oxidizing it [18] or using atomic layer deposition (ALD) techniques [19]. The channel mobility, saturation velocity, and transport characteristics of MOS-HEMTs with Al_2_O_3_ as the gate dielectric have been reported to be higher than those of conventional HEMT devices. A reported Al_2_O_3_ MOS-HEMT operating at 4 GHz demonstrated an output power density of 5.76 W/mm and a power-added efficiency (PAE) of 57% [20]. Additionally, MOS-HEMTs combining higher dielectric constant HfO_2_ and Al_2_O_3_ with good interface properties have been developed, showing excellent frequency characteristics. Including a superior subthreshold slope (*S*.*S*.) of 70 mV/dec and a high drain–source current (*I_DS_*) on–off ratio of up to six orders, the cutoff frequency (f_T_) and maximum oscillation frequency (f_max_) reached 21.1 GHz and 38.9 GHz, respectively [21].

In this paper, we propose a new enhancement-mode HEMT with a dual Al_2_O_3_ MIS gate structure to promote DC and RF characteristics. The Al_2_O_3_ MIS microstructure could be made in a planar form and recessed form. Utilizing Silvaco TCAD 2024 version and Victory Process 8.42.2.R, simulations were performed to analyze the transfer characteristics and output characteristics of the devices. The superiority of the dual Al_2_O_3_ MIS gate structure was compared and verified. Additionally, the two-dimensional electron gas distribution and the band energy were simulated, explaining the reasons for parameter optimization from a physical perspective. Furthermore, it was found that the C–V characteristic exhibits a hysteresis effect in newly designed structures. The AC characteristics of the three structures were further investigated, with a focus on parameters such as the cutoff frequency, maximum oscillation frequency, and minimum noise figure.

## 2. Device Structures and Simulation Parameters

**Figure 1:**
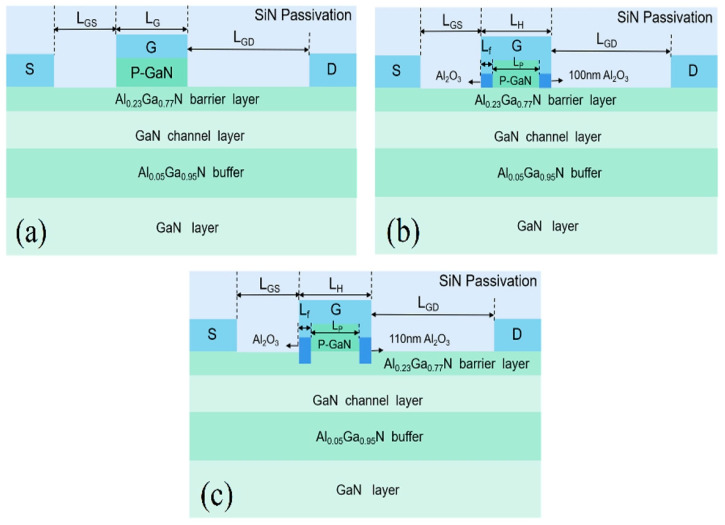
Schematic cross-section of (**a**) traditional HEMT [22], (**b**) SC-MOS HEMT, and (**c**) SCR-MOS HEMT.

Figure 1 shows the cross-sectional schematic diagram of the conventional p-GaN enhancement-mode HEMT, the HEMT with a dual Al_2_O_3_ MIS gate structure and shortened cap layer (SC-MOS HEMT), and the former HEMT with a recessed dual Al_2_O_3_ MIS gate structure (SCR- MOS HEMT). The conventional structure can be manufactured according to the HEMTs produced by O. Hilt et al. [22], which consists of a 15 nm undoped Al_0.23_Ga_0.77_N barrier layer, 35 nm GaN channel layers, and a 2 μm Al_0.05_Ga_0.95_N buffer layer grown by MOCVD. The device utilizes the 600 μm Si as the substrate and a 50 nm thick AlN nucleation layer is grown on it by magnetron sputtering. Then a 110 nm thick p-type GaN layer is grown on the AlGaN barrier in order to facilitate the normally-off operation, with a magnesium doping concentration of 3 × 10^17^ cm^−3^. In the traditional structure, the length of the P-GaN cap is the same as the gate length, which is 1.4 μm. In the SC-MOS HEMT structure, the length of the P-GaN cap (LP) is reduced to 1 μm, and two 100 nm thick Al_2_O_3_ layers are added beneath the gate feet on both sides of the P-GaN. In the two proposed structures, the length of the gate head (L_H_) is 1.4 μm and the length of the gate foot (Lf) on both sides is 0.2 μm. Compared to the SC-MOS HEMT, the Al_2_O_3_ layer in the SCR-MOS HEMT has a thickness of 110 nm, with 10 nm Al_2_O_3_ recessed in the barrier layer. The relevant device parameters are listed in Table 1.

**Figure 2:**
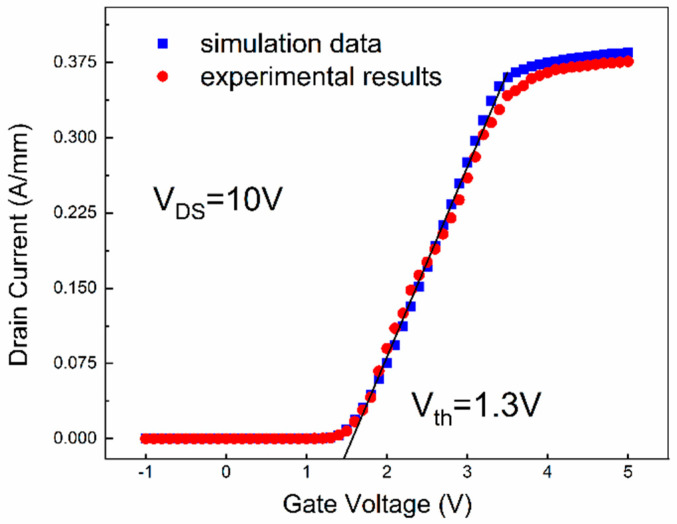
Transfer characteristic curves of simulation data and experimental results.

Two-dimensional numerical simulations are operated and analyzed by using Silvaco TCAD. According to the report of Eldad Bahat [23], 2DEG induced by piezoelectric polarization and the spontaneous effect is specified in the parameters of piezoelectric constants and elastic constants (E_31_ E_33_ C_31_ C_33_), which can be seen in Table 2. In addition, the lattice constant (a_0_) is set as the average value. The Shockley–Read–Hall model is used for carrier recombination simulation while the k.p model is adopted for drift-diffusion in the simulation. Also, the Faramand Modified Caughey Thomas model and Nitride Field Dependent model are also used for electron and hole mobility [24,25]. Simultaneously, the donor trap level is fixed at 2.2 eV below the conduction band and the acceptor trap level is fixed at 0.36 eV above the valence band, with a density of 1.27 × 10^18^ cm^−3^ and 7 × 10^17^ cm^−3^, respectively. In order to simulate the surface states of the device, the state densities at the Al_2_O_3_/AlGaN interface are set to 1 × 10^12^ cm^−2^ eV^−1^. Finally the simulated transfer characteristic curves and the corresponding experimental data are compared in Figure 2. The good agreement between the numerical results and the experimental results indicates the models used in the simulation are effective and reliable.

## 3. Results and Discussion

### 3.1. DC Performance

**Figure 3:**
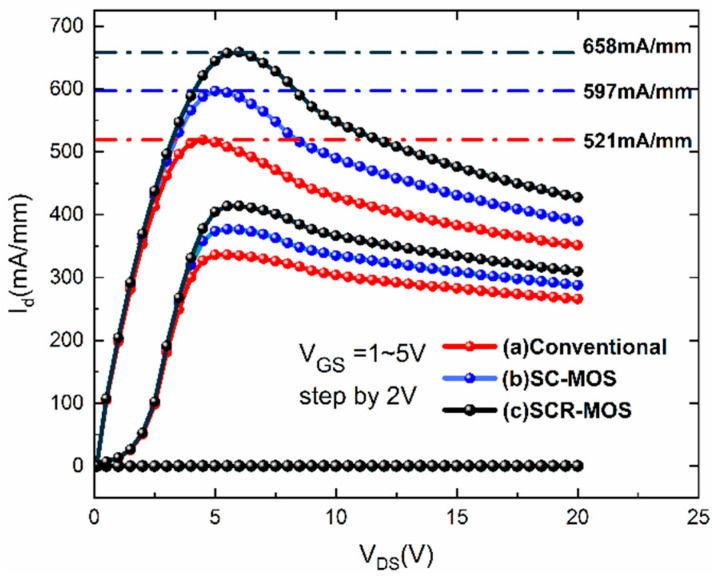
Output characteristic curves as functions of drain voltage in three structures.

Figure 3 clearly shows an increase in drain current. When the gate–source voltage (V_GS_) is below the threshold voltage (1.5 V), the device is in the off state and the drain current is 0. As V_GS_ remains constant and the drain voltage increases, the drain current initially grows rapidly and tends to be saturated since the drain voltage reaches the knee voltage. Due to the self-heating effect set in the simulation [26], the drain current declines gradually. The maximum saturation drain current (I_DSmax_) for the traditional HEMT, SC-MOS HEMT, and SCR-MOS HEMT structures are 519 mA/mm, 597 mA/mm, and 658 mA/mm, respectively. The enhancement in I_DSmax_ could be attributed to the shortened p-GaN cap layer and the recessed Al_2_O_3_ microstructure. The shortened p-GaN length allows less 2DEG to be depleted and the conductive channel could open more easily. In addition, the suppression of the barrier interface charge and channel carrier scattering facilitates the higher I_DSmax_ in the SC-MOS HEMT and SCR-MOS HEMT, which is brought by the dual recessed Al_2_O_3_ MIS gate structure.

**Figure 4:**
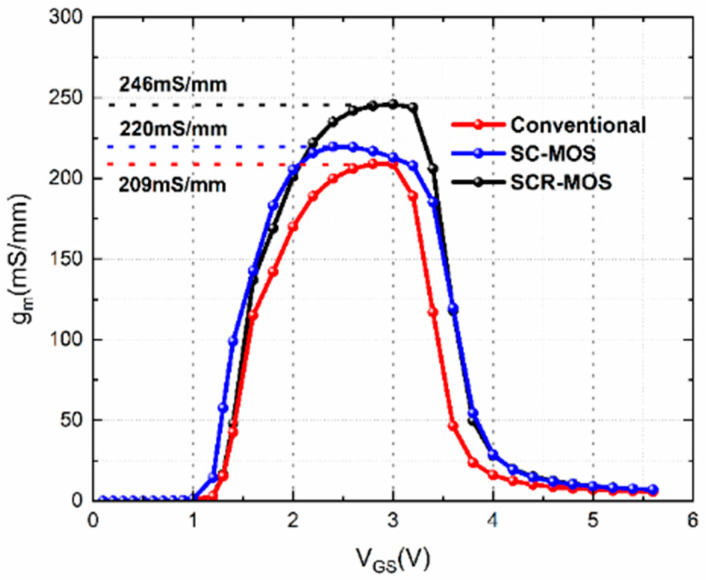
Transconductance curves as functions of gate voltage in three structures at V_DS_ = 15 V.

Figure 4 shows the curve of transconductance (g_m_) as a function of gate–source voltage for the three structures. The peak transconductance is 209 mS/mm, 220 mS/mm, and 246 mS/mm for the traditional HEMT, SC-MOS HEMT, and SCR-MOS HEMT, respectively. The HEMTs with the MIS microstructure not only own a higher peak, but the g_m_ range is also larger than that of traditional devices. The shorter p-GaN length and dielectric connecting gate make the gate closer to the conductive channel and boost gate control capacity.

**Figure 5:**
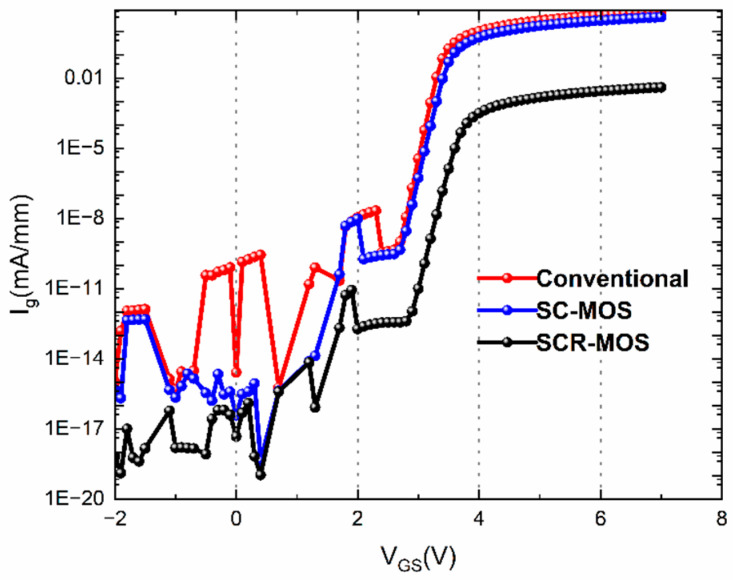
Gate leakage current density as function of gate voltage at V_DS_ = 15 V.

In the off-state gate leakage current curves illustrated in Figure 5, the off-state gate leakage current in the SCR-MOS HEMT represents an obvious reduction from 2.58 × 10^−15^ A·mm^−1^ to 4.73 × 10^−18^ A·mm^−1^, which means the I_on_/I_off_ ratio has improved three orders of magnitude compared to the traditional HEMT and SC-MOS HEMT. The lower off-state gate current indicates better 2DEG depletion brought by the recessed dielectric and its superiority in promoting device reliability and breakdown voltage. In the device’s on-state, the gate current of the SCR HEMT is also significantly smaller than that of the other two structures. The introduction of the dielectric increases the gate capacitance’s ability to control the gate charge, which not only enhances the transconductance but also ensures a small gate leakage current, thereby better ensuring the device’s high output current density.

### 3.2. AC Performance

**Figure 6:**
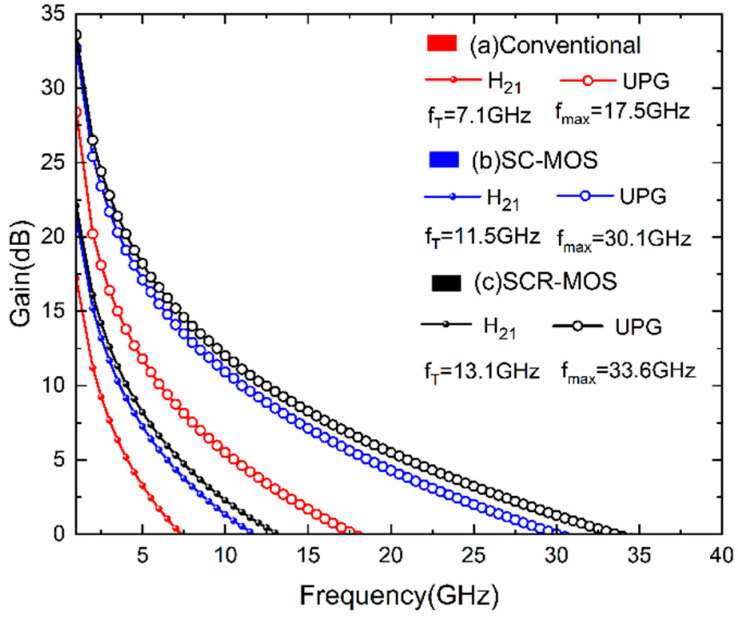
Current gain and unilateral power gain versus frequency at V_DS_ = 15 V and V_GS_ = 3 V.

As shown in Figure 6, the curves of current gain and unilateral power gain versus frequency are presented for the three structures, with the drain voltage fixed at 15 V and the gate voltage set to 3 V. The cutoff frequency (f_T_) for the traditional structure is 7.1 GHz, and the maximum oscillation frequency (f_max_) is 17.5 GHz. In the SC-MOS HEMT, f_T_ reaches 11.5 GHz and f_max_ increases to 30.1 GHz. Further embedding the Al_2_O_3_ layer into the barrier layer raises f_T_ to 13.1 GHz and f_max_ to 33.6 GHz.

**Figure 7:**
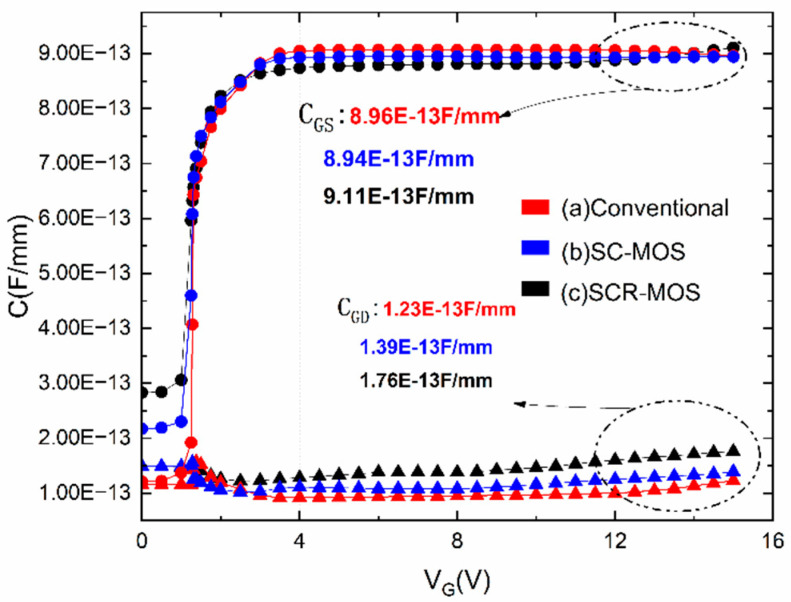
Gate–source capacitance and gate–drain capacitance versus V_GS_ at V_DS_ = 15 V and frequency = 1 GHz.

To investigate the reason for the enhanced frequency performance, Figure 7 shows the variation of the gate–source capacitance and gate–drain capacitance with gate voltage for the three structures, with the frequency fixed at 1 GHz and the source–drain voltage fixed at 15 V. The maximum values of gate–source capacitance (C_gs_) for the three structures are 0.89 pF/mm (conventional), 0.89 pF/mm (SC-MOS HEMT), and 0.91 pF/mm (SCR-MOS HEMT), while the maximum values of gate–drain capacitance (C_gd_) are 0.12 pF/mm (conventional), 0.14 pF/mm (SC-MOS HEMT), and 0.17 pF/mm (SCR-MOS HEMT). Although Al_2_O_3_ possesses a high permittivity compared to SiN, the relatively small width of the added Al_2_O_3_ layer only slightly increases C_gs_ and C_gd_ and the influence is small enough to be negligible.

**Figure 8:**
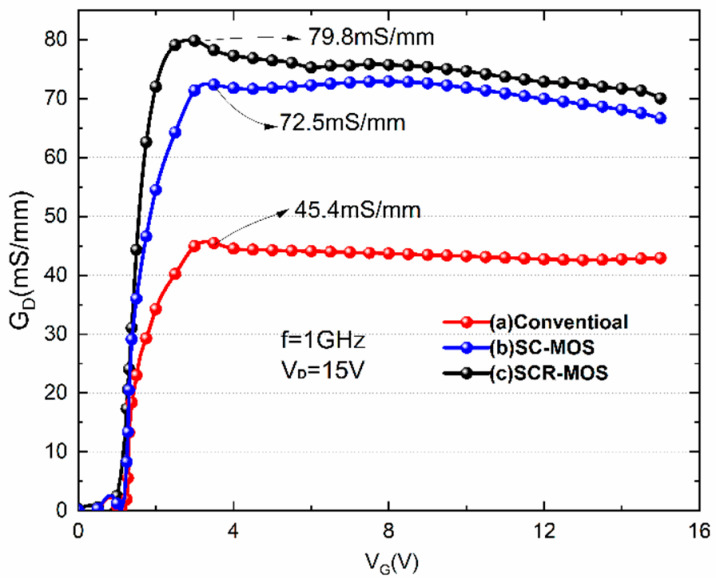
Gate–drain conductance versus V_GS_ at V_DS_ = 15 V and frequency = 1 GHz.

In Figure 8, the gate–drain conductance is shown as the functions of gate voltage, with the frequency fixed at 1 GHz and the drain voltage fixed at 15 V. The traditional structure has the smallest conductance of 45.4 mS/mm, indicating that the drain series resistance (R_D_) in the traditional structure is relatively large. The conductance of the non-recessed Al_2_O_3_ structure is 72.5 mS/mm, and the conductance of the recessed Al_2_O_3_ structure is 79.8 mS/mm. The successive increase in conductance for the three structures indicates a decrease in their inverse resistances.

**Figure 9:**
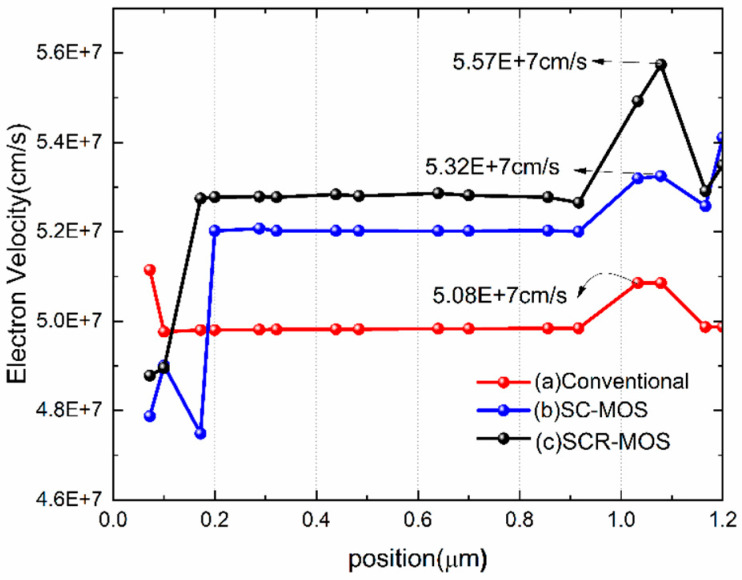
Electron velocity beneath the gate at V_DS_ = 15 V and V_GS_ = 3 V.

The schematic of the saturated electron velocity with the position beneath the gate is shown in Figure 9. In the SC-MOS HEMT and SCR-MOS HEMT, the electron saturation velocity is increased to 5.32 × 10^7^ cm/s and 5.57 × 10^7^ cm/s, compared to 5.08 × 10^7^ cm/s in the traditional HEMT. The introduction of the MIS microstructure suppresses part of the surface charge and inhibits carrier scattering and oscillation, which is beneficial to electron mobility and saturation velocity.

### 3.3. Physical Insight and Optimization

**Figure 10:**
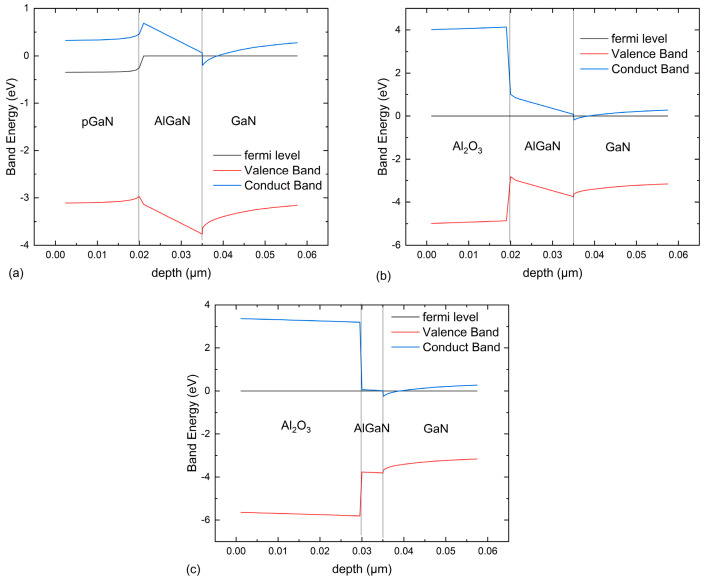
Comparison of the band diagrams for the (**a**) traditional HEMT (**b**) SC-MOS HEMT, and (**c**) SCR-MOS HEMT.

Figure 10 shows the band diagrams for three different structures, taken from the position of the MIS gate structure. The gate voltage bias is set to 3 V to ensure the device is in the “on” state, while the drain is in an unbiased state. It can be seen that in the traditional structure, the bandgap of p-GaN is relatively small, making it difficult to form a high barrier. The electrons in the channel have a higher chance of transitioning to p-GaN, resulting in a larger gate leakage current. The bandgap and dielectric constant of Al_2_O_3_ are larger, so the barrier beneath the gate of the SC-MOS HEMT and SCR-MOS HEMT is higher, making it more difficult for the channel electrons to transition, thus ensuring a lower gate leakage current. Additionally, the contact between Al_2_O_3_ and AlGaN alters the energy band. For the SCR-MOS structure, the 2DEG potential well depth is 0.25 eV, which is higher than the 0.18 eV in the SC-MOS and the 0.2 eV in the traditional structure. A deeper 2DEG potential well means a higher 2DEG concentration, thereby improving the device’s output current.

**Figure 11:**
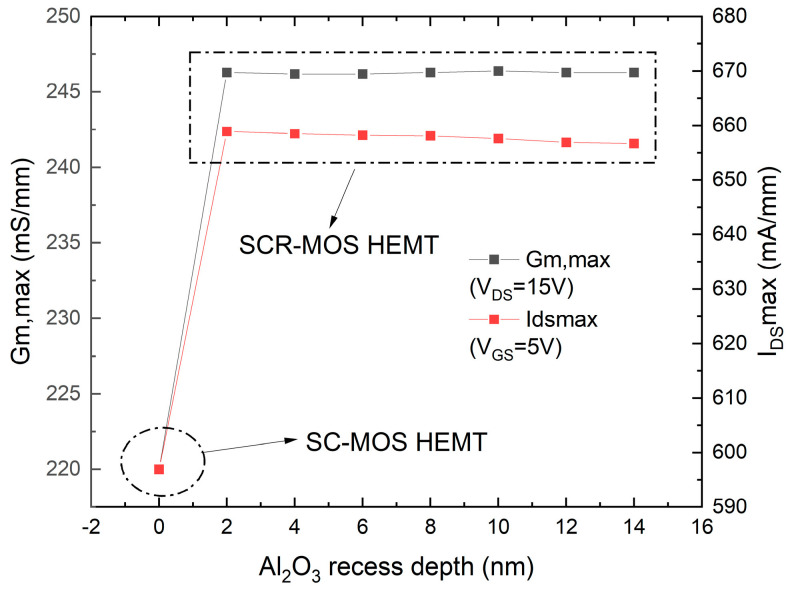
Maximum transconductance and maximum saturation drain current versus different Al_2_O_3_ recess depth.

**Figure 12:**
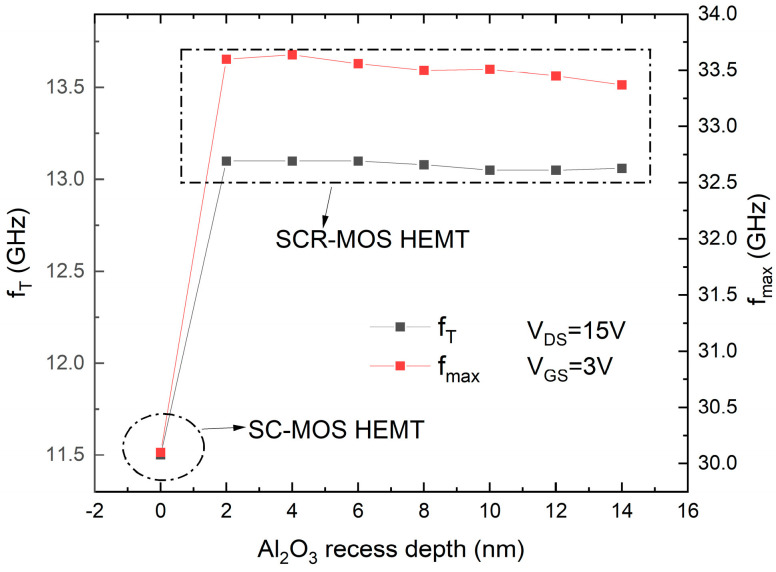
Cutoff frequency and maximum oscillation frequency versus different Al_2_O_3_ recess depth.

To further optimize the device design, the recessed depth of Al_2_O_3_ is adjusted to determine the optimal device performance. As shown in Figure 11, the impact of changing the recessed depth of Al_2_O_3_ on the device’s g_m,max_ and I_DSmax_ is demonstrated. The recessed depth of zero corresponds to the SC-MOS structure. It can be seen that the recessed depth of Al_2_O_3_ has little effect on the device’s g_m,max_ and I_DSmax_. As the recessed depth increases, the transconductance peak remains almost constant at 246 mS/mm, indicating that the gate control capability of the device remains unchanged. The I_DSmax_ gradually decreases from 658 mA/mm to a minimum of 656 mA/mm, indicating that a greater recessed depth makes it easier to affect the carrier migration in the channel, slightly decreasing the output current. Figure 12 shows the effect of changing the recessed depth of Al_2_O_3_ on the device’s f_T_ and f_max_. Similarly, the recessed depth of Al_2_O_3_ has little impact on the device’s ft and fmax. As the recessed depth increases, f_T_ decreases from a maximum of 13.1 GHz to a minimum of 13.05 GHz, and f_max_ decreases from 33.6 GHz to 33.37 GHz.

**Figure 13:**
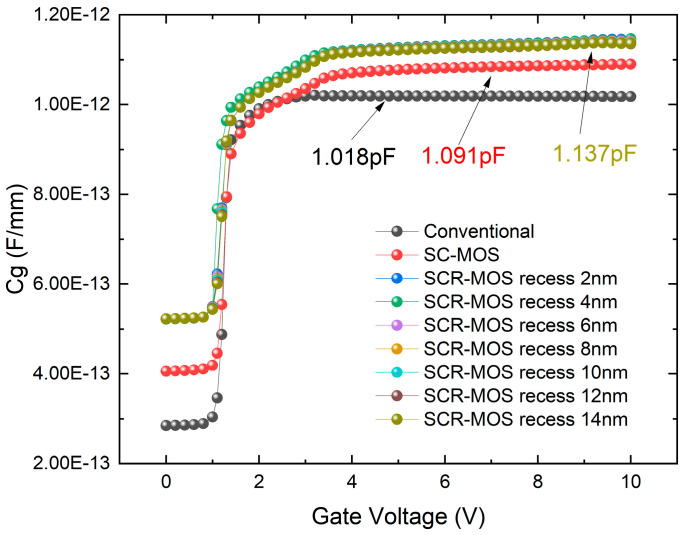
Gate capacitance versus V_GS_ at V_DS_ = 15 V and frequency = 1 GHz.

The gate capacitance (C_g_) of the three structures was also studied in Figure 13. It can be seen that the C_g_ of the SC-MOS structure and SCR-MOS structure is larger than the traditional structure’s 1.018 pF, with the Cg of the SCR-MOS structure being larger than the 1.091 pF of the SC-MOS structure. Due to the high dielectric constant of Al_2_O_3_, an increase in capacitance is expected. A larger gate capacitance helps increase the gate control capability of the device and reduces the generation of the gate leakage current. As the recessed depth of Al_2_O_3_ increases, C_g_ gradually decreases from 1.146 pF to 1.137 pF, with only a slight change. After ensuring that the SCR-MOS structure has better DC and RF performance, the gate capacitance should be as small as possible to prevent large gate capacitance from reducing the device’s high-frequency response speed. Combining Figure 11 and Figure 12, a recessed depth of 8 nm for Al_2_O_3_ provides the best performance.

There are two equations to express f_T_ and f_max_ as follows, where R_I_ is the intrinsic input resistance, R_ds_ is the drain–source series resistance, and R_S_, R_D_, and R_G_ are the source resistance, drain resistance, and gate resistance, respectively.

(1) $$f_{T}=\frac{g_{m}/2\pi }{\left(C_{\mathrm{gs}}+{\;C}_{\mathrm{gd}}\right)\left[1+\left(R_{S}+R_{D}\right)/R_{\mathrm{ds}}\right]+C_{\mathrm{gd}}g_{m}(R_{S}+{\;R}_{D})}$$

(2) $$f_{\max}=\frac{f_{T}}{2\sqrt{\left(R_{G}+{\;R}_{S}+R_{I}\right)/R_{\mathrm{ds}}+2\pi f_{T}R_{G}C_{\mathrm{gd}}}}$$

Typically, R_D_ could be expressed as the sum of the source–drain ohmic contact resistance and the conduction resistance in Equation (3). As the sheet resistance, R_SH_ could be further shown as Equation (4):

(3) $$R_{D}=R_{C}+{\;R}_{\mathrm{SH}}\frac{L_{\mathrm{GD}}}{W}$$

(4) $$R_{\mathrm{SH}}=\frac{1}{\mu {\mathrm{en}}_{s2D}}$$

where μ is the mobility, which is primarily determined by the material properties. As analyzed earlier, the new two structures have a higher mobility compared to the conventional structure, which means a lower R_SH_. ${en}_{s2D}$ represents the 2DEG concentration related to the barrier layer thickness and the threshold voltage, which can be explained by the following formula:

(5) $${\mathrm{en}}_{s2D\;}={\;C}_{1}(V_{G}\;-{\;V}_{T})$$

(6) $$C_{1}=\varepsilon_{1}/(d+∆d)\;\approx {\;\varepsilon }_{1}/d$$

where $\varepsilon_{1}$ is the dielectric constant of the AlGaN side in the AlGaN/GaN heterojunction, and $C_{1}$ is the unit area capacitance between the gate and the channel. The distance ∆d between the 2DEG and the AlGaN/GaN heterojunction interface can be neglected compared to the barrier layer thickness d. In the three structures, the addition of the high permittivity Al_2_O_3_ increases the average dielectric constant on the AlGaN side. The recessed Al_2_O_3_ layer further reduces the average thickness of the AlGaN barrier layer. As a result, from Equation (6), the unit capacitance between the gate and the channel increases, allowing more charge to be stored. This also explains why the current increases after adding the Al_2_O_3_ layer and the decrease in R_D_. Similarly, both R_S_ and R_ds_ are also diminished. On the basis of enhanced transconductance (g_m_) before and reduced resistance, it is possible to obtain the enhanced f_T_ and f_max_ in the newly designed structure.

**Figure 14:**
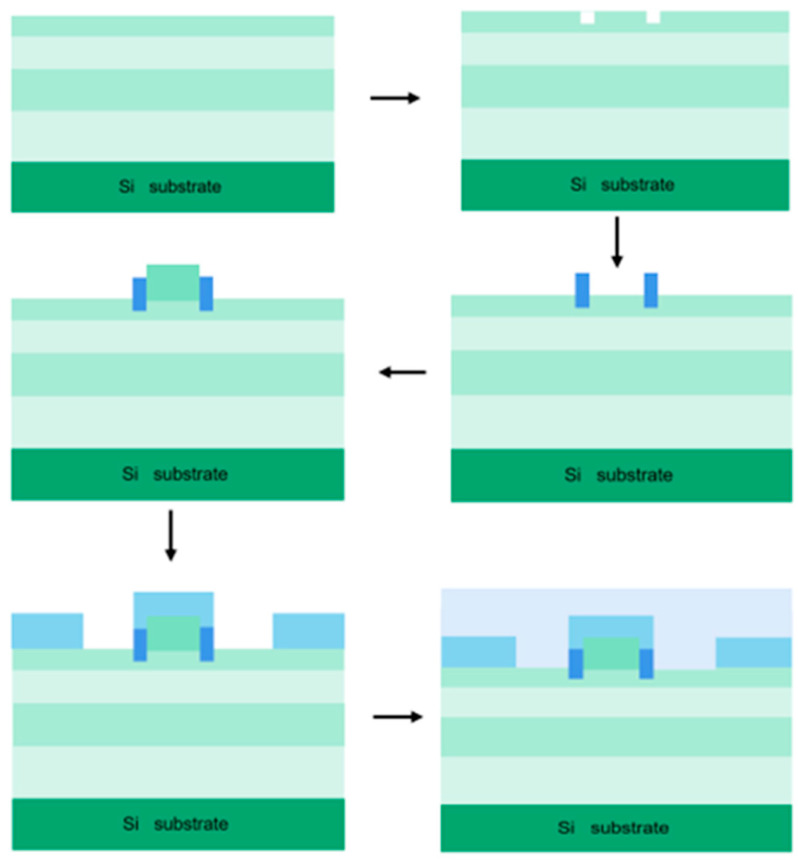
Process flow diagram of AlGaN/GaN HEMT with aluminum oxide MIS gate structure.

The process flow diagram for the SCR-MOS HEMT is shown in Figure 14. Using MOCVD, epitaxial growth is performed on a 2-inch (111) crystal-oriented silicon substrate. From bottom to top, the GaN buffer layer, 2 μm Al_0.05_Ga_0.95_N back-surface layer, 35 nm GaN channel layer, and 15 nm Al_0.23_Ga_0.77_N barrier layer are grown. Photolithography is then used to etch the MIS region on the 15 nm Al_0.23_Ga_0.77_N barrier layer. A groove with a width of 0.2 μm and a depth of 8 nm is etched at the edge of the gate exposure alignment area. Aluminum oxide is deposited in the etched region using CVD, with a deposition thickness of 108 nm. Next, a 110 nm thick GaN cap layer is grown between the two aluminum oxide dielectric layers on the unetched Al_0.23_Ga_0.77_N barrier layer surface. Magnesium ions with a concentration of 3 × 10^17^ cm^−3^ are implanted to form a P-type doped GaN layer. The area above the aluminum oxide dielectric, with a width of 110 nm, is the gate foot region. The region above the P-GaN cap layer, with a width of 1.4 μm and thickness of 100 nm, is the gate cap region. Ti/Al/Ni/Au is deposited as the gate electrode using electron beam evaporation. Ohmic contact electrodes are formed through electron beam evaporation to create the drain and source electrodes, followed by thermal annealing at 830 °C in a nitrogen atmosphere. Finally, a 240 nm thick Si_3_N_4_ passivation layer is deposited to cover the entire electrode.

## 4. Conclusions

This paper improves the structure of the traditional AlGaN/GaN enhancement-mode HEMT to achieve better DC and AC performance, making it more suitable for applications in power and RF fields. With a dual Al_2_O_3_ MIS gate structure, the proposed new structures increase the maximum drain–source current from the original 519 mA/mm to 658 mA/mm (a 26% improvement), the transconductance from 209 mS/mm to 246 mS/mm, and reduces the gate leakage current by three orders of magnitude when the device is in the off state. The cutoff frequency of the proposed structure increases by 84% (from 7.1 GHz to 13.1 GHz), the maximum oscillation frequency increases by 92% (from 17.5 GHz to 33.6 GHz), and the current gain improves by 30% (from 17.3 dB to 22.5 dB). These results indicate that AlGaN/GaN devices with a dual Al_2_O_3_ MIS gate structure exhibit excellent RF performance.

## Figures and Tables

**Table 1:** Main device parameters for the simulation.

Symbol	Statement	Value
LGS	Gate–source distance	1.1 μm
LGD	Gate–drain distance	6 μm
LG&LH	Length of gate and length of p-GaN in traditional HEMT	1.4 μm
LP	Length of p-GaN in SC-MOS HEMT and SCR-MOS HEMT	1 μm
Lf	Length of gate foot	0.2 μm
TP	Thickness of p-GaN	110 nm
Tb	Thickness of AlGaN barrier	15 nm
Ho	Height of oxide above barrier layer	100 nm
Do	Depth of oxide recessed in barrier layer	10 nm

**Table 2:** Key parameters set in simulation model.

Parameter	Symbol	Value
Lattice constant	a_0_	3.189 Å
Elastic constant	C_33_	392 Gpa
Elastic constant	C_13_	100 Gpa
Spontaneous polarization	P_sp_	−0.034 C·m^−2^
Piezoelectric const.(z)	E_33_	0.68 C·m^−2^
Piezoelectric const.(x,y)	E_31_	−0.32 C·m^−2^

## Data Availability

The original contributions presented in this study are included in the article. Further inquiries can be directed to the corresponding author.

## References

[1] Brinkhoff J., Parker A.E. Characterising HEMT devices for MMIC distortion prediction. Proceedings of the SPIE Conference Proceedings.

[2] Iucolano F., Boles T. (2019). GaN-on-Si HEMTs for wireless base stations. Mater. Sci. Semicond. Process..

[3] Lee S.-Y., Woo J., Park S., Kwon Y. (2016). Linear X-band GaN HEMT transformer-based Doherty power amplifier. Electron. Lett..

[4] Wu T.-L., Marcon D., You S., Posthuma N., Bakeroot B. (2015). Forward Bias Gate Breakdown Mechanism in Enhancement-Mode p-GaN Gate AlGaN/GaN High-Electron Mobility Transistors. Electron Device Lett..

[5] Wang H., Wei J., Xie R., Liu C., Tang G., Chen K.J. (2016). Maximizing the Performance of 650-V p-GaN Gate HEMTs: Dynamic RON Characterization and Circuit Design Considerations. IEEE Trans. Power Electron..

[6] Medjdoub F., Waldhoff N., Zegaoui M., Grimbert B., Rolland N., Rolland P.A. (2011). Low-Noise Microwave Performance of AlN/GaN HEMTs Grown on Silicon Substrate. IEEE Electron Device Lett..

[7] Allaei M., Shalchian M., Jazaeri F. (2020). Modeling of short-channel effects in GaN HEMTs. IEEE Trans. Electron Devices.

[8] Saadaoui S., Fathallah O., Maaref H. (2022). Effects of gate length on GaN HEMT performance at room temperature. J. Phys. Chem. Solids.

[9] Moultif N., Latry O., Ndiaye M., Neveu T., Joubert E., Moreau C., Goupy J.-F. (2019). S-band pulsed-RF operating life test on AlGaN/GaN HEMT devices for radar application. Microelectron. Reliab..

[10] Zanoni E., Santi C.D., Gao Z., Buffolo M., Fornasier M., Saro M., Pieri F.D., Rampazzo F., Meneghesso G., Meneghini M. (2024). Microwave and millimeter-wave GaN HEMTs: Impact of epitaxial structure on short-channel effects, electron trapping, and reliability. IEEE Trans. Electron Devices.

[11] Guan H., Li W., Tong X., Shen G., Li F., Zhang H. (2023). DC and RF performance of HR Si(111)-based AlGaN/GaN MIS-HEMT with a symmetrical multi-finger grid array structure for 5G N28 700MHz low-bias-control applications. Mater. Sci. Semicond. Process..

[12] Kim J.-G. (2024). Optimization of epitaxial structures on GaN-on-Si (111) HEMTs with step-graded AlGaN buffer layer and AlGaN back barrier. Coatings.

[13] Dutta G., DasGupta N., DasGupta A. (2017). Gate Leakage Mechanisms in AlInN/GaN and AlGaN/GaN MIS-HEMTs and Its Modeling. IEEE Trans. Electron Devices.

[14] Yagi S., Shimizu M., Inada M., Yamamoto Y., Piao G., Okumura H., Yano Y., Akutsu N., Ohashi H. (2006). High breakdown voltage AlGaN/GaN MIS–HEMT with SiN and TiO2 gate insulator. Solid-State Electron..

[15] Tang Z., Jiang Q., Lu Y., Huang S., Yang S., Tang X. (2013). 600-V Normally Off SiNx/AlGaN/GaN MIS-HEMT With Large Gate Swing and Low Current Collapse. IEEE Electron Device Lett..

[16] Liu S., Yang S., Tang Z., Jiang Q., Liu C., Wang M., Chen K.J. (2014). Al_2_O_3_/AlN/GaN MOS-Channel-HEMTs With an AlN Interfacial Layer. IEEE Electron Device Lett..

[17] Mondal A., Roy A., Mitra R., Kundu A. (2020). Comparative Study of Variations in Gate Oxide Material of a Novel Underlap DG MOS-HEMT for Analog/RF and High Power Applications. Silicon.

[18] Chen M., Xu J., Cao Y., He H.-Y., Fan K.N., Zhuang H. (2010). Dehydrogenation of propane over In_2_O_3_–Al_2_O_3_ mixed oxide in the presence of carbon dioxide. J. Catal..

[19] Boryło P., Lukaszkowicz K., Szindler M., Kubacki J., Balin K., Basiaga M., Szewczenko J. (2016). Structure and properties of Al_2_O_3_ thin films deposited by ALD process. Vacuum.

[20] Huang S., Liu X., Zhang J., Wei K., Liu G., Wang X., Zheng Y., Liu H., Jin Z., Zhao C. (2015). High RF Performance Enhancement-Mode Al_2_O_3_/AlGaN/GaN MIS-HEMTs Fabricated with High-Temperature Gate-Recess Technique. IEEE Electron Device Lett..

[21] Lee C.-S., Liao Y.-H., Chou B.-Y., Liu H.-Y., Hsu W.-C. (2014). Composite HfO_2_/Al_2_O_3_-dielectric AlGaAs/InGaAs MOS-HEMTs by using RF sputtering/ozone water oxidation. Superlattices Microstruct..

[22] Hilt O., Knauer A., Brunner F., Treidel E.B., Würfl J. Normally-off AlGaN/GaN HFET with p-type GaN gate and AlGaN buffer. Proceedings of the 6th International Conference on Integrated Power Electronics Systems.

[23] Treidel E.B., Hilt O., Brunner F., Sidorov V., Würfl J., Tränkle G. (2010). AlGaN/GaN/AlGaN DH-HEMTs Breakdown Voltage Enhancement Using Multiple Grating Field Plates (MGFPs). IEEE Trans. Electron Devices.

[24] Oğuzman I.H., Bellotti E., Brennan K.F., Kolník J., Wang R., Ruden P.P. (1997). Theory of hole initiated impact ionization in bulk zincblende and wurtzite GaN. J. Appl. Phys..

[25] Mnatsakanov T.T., Levinshtein M.E., Pomortseva L.I., Yurkov S.N., Simin G.S., Khan M.A. (2003). Carrier mobility model for GaN. Solid-State Electron..

[26] Gryglewski D., Wojtasiak W., Kamińska E., Piotrowska A. (2020). Characterization of Self-Heating Process in GaN-Based HEMTs. Electronics.

